# Two-Dimensional Shear-Wave Elastography: Accuracy in Liver Fibrosis Staging Using Magnetic Resonance Elastography as the Reference Standard

**DOI:** 10.3390/diagnostics15010062

**Published:** 2024-12-29

**Authors:** Puwitch Charoenchue, Jiraporn Khorana, Taned Chitapanarux, Nakarin Inmutto, Wittanee Na Chiangmai, Amonlaya Amantakul, Suwalee Pojchamarnwiputh, Apichat Tantraworasin

**Affiliations:** 1 Department of Radiology, Faculty of Medicine, Chiang Mai University, Chiang Mai 50200, Thailand; puwitch.c@cmu.ac.th (P.C.); nakarin.i@cmu.ac.th (N.I.); wittanee.n@cmu.ac.th (W.N.C.); amonlaya.amantakul@cmu.ac.th (A.A.); 2 Department of Surgery, Faculty of Medicine, Chiang Mai University, Chiang Mai 50200, Thailand; jiraporn.kho@cmu.ac.th; 3 Department of Biomedical Informatics and Clinical Epidemiology, Faculty of Medicine, Chiang Mai University, Chiang Mai 50200, Thailand; 4 Clinical Surgical Research Center, Faculty of Medicine, Chiang Mai University, Chiang Mai 50200, Thailand; 5 Department of Internal Medicine, Faculty of Medicine, Chiang Mai University, Chiang Mai 50200, Thailand; taned.chi@cmu.ac.th

**Keywords:** 2D shear-wave elastography, magnetic resonance elastography, liver fibrosis, noninvasive diagnosis, chronic liver disease

## Abstract

**Background:** The accurate staging of liver fibrosis is crucial for managing chronic liver disease (CLD). Although magnetic resonance elastography (MRE) is the reference standard for noninvasive fibrosis assessment, its cost, specialized hardware, and operational demands restrict accessibility. In contrast, two-dimensional shear-wave elastography (2D-SWE) is more affordable, accessible, and widely integrated into routine ultrasound systems. **Objective:** Our aim was to determine the optimal 2D-SWE cut-offs for detecting significant fibrosis (≥F2) and evaluate its diagnostic performance across fibrosis stages. **Methods:** In this prospective study, 71 patients with suspected CLD underwent same-day MRE and 2D-SWE. MRE-defined cut-offs categorized fibrosis stages (≥3.5 kPa for significant fibrosis). Sensitivity, specificity, area under the receiver operating characteristic curve (AUROC), and likelihood ratios were calculated for various 2D-SWE thresholds. **Results:** At a 2D-SWE cut-off of 7.0 kPa, sensitivity for detecting ≥F2 fibrosis was 100% with a specificity of 85.7% and a positive likelihood ratio (LR+) of 7.0. Increasing the threshold to 8.0 kPa improved specificity to 91.8% while maintaining a sensitivity of 86.4% and achieving an AUROC of 0.89. For cirrhosis, a cut-off of 11.0 kPa achieved 100% sensitivity and 96.9% specificity. A 5.0 kPa cut-off reliably excluded abnormal stiffness with 89.1% sensitivity. **Conclusions:** Two-dimensional SWE is a reliable method for staging liver fibrosis. Thresholds of 7.0 kPa for screening significant fibrosis, 8.0 kPa for confirmation, and 11.0 kPa for diagnosing cirrhosis demonstrate high diagnostic accuracy. A 5.0 kPa cut-off effectively excludes abnormal liver stiffness.

## 1. Introduction

Chronic liver disease (CLD) is a significant global health issue responsible for considerable morbidity and mortality worldwide. The progressive development of liver fibrosis, a hallmark of CLD, increases the risk for complications like cirrhosis and hepatocellular carcinoma, making accurate fibrosis staging crucial for effective management. Globally, cirrhosis and liver cancer account for over two million deaths annually, underscoring the importance of timely and accurate disease assessment [1,2].

Liver biopsy has traditionally served as the gold standard for assessing liver fibrosis due to its direct histopathological insights. However, biopsy is invasive, associated with potential risks such as bleeding and infection, and is limited by sampling variability since it captures only a small tissue sample [3,4]. Additionally, biopsy reliability can be influenced by operator expertise, and variability between samples may lead to staging inconsistencies [5,6].

Magnetic resonance elastography (MRE) has gained recognition as a highly accurate noninvasive alternative to biopsy, offering full-liver stiffness maps and minimizing patient risk. Studies show that MRE achieves comparable accuracy to biopsy in staging liver fibrosis, with advantages of broader coverage and reduced sampling error [3,7,8,9,10]. MRE’s high sensitivity and specificity have been consistently validated across various etiologies of CLD, and research supports its reliability in diagnosing and staging fibrosis without the invasiveness of biopsy [11,12,13,14,15,16]. Furthermore, MRE’s ability to evaluate fibrosis over the entire liver volume rather than a small sample enhances diagnostic precision and reproducibility, making it an ideal reference standard in noninvasive fibrosis evaluation [14,17,18].

Transient elastography (TE), commonly performed using devices such as FibroScan^®^, is a well-established, noninvasive technique for liver stiffness measurement. While TE demonstrates high accuracy in detecting advanced fibrosis and cirrhosis, its performance may be compromised in patients with obesity or ascites due to technical limitations in wave propagation and measurement reliability [19]. Additionally, TE requires specialized equipment that is not always accessible in routine settings [20]. Although TE offers practicality and moderate accuracy, these constraints highlight the need for versatile, accessible alternatives.

Shear-wave elastography (SWE) is an ultrasound-based method that integrates with conventional ultrasound systems, broadening its availability. SWE allows real-time liver stiffness measurements and has demonstrated diagnostic accuracy comparable to that of TE for liver fibrosis staging, particularly in chronic liver disease contexts [21,22]. Recent studies suggest SWE’s accuracy in correlating liver stiffness values with fibrosis stages, making it a promising tool for fibrosis assessment in diverse clinical settings [23,24].

MRE is recognized as a reference standard for noninvasive liver fibrosis staging due to its high diagnostic accuracy and ability to assess the entire liver [25]. However, its widespread use is limited by specific challenges. MRE requires specialized hardware, including external drivers and advanced imaging software, in addition to trained technologists and radiologists for operation and interpretation [26]. Moreover, the technique is resource-intensive, time-consuming, and associated with high costs, even in centers with MRI capabilities. These constraints restrict its availability globally, particularly in settings with limited healthcare resources. Such barriers highlight the need for alternative noninvasive tools, such as 2D-SWE, which are more cost-effective, accessible, and easily integrated into conventional ultrasound systems, to improve access to liver fibrosis staging across diverse healthcare environments.

The primary objective of this study was to determine the optimal SWE cut-off value for identifying significant fibrosis (≥F2), a critical threshold for clinical decision making in chronic liver disease. Identifying this cut-off is essential for detecting patients at higher risk of disease progression and complications. While the accuracy of SWE across other fibrosis stages (F1, F3, and F4) were also assessed, these findings served as secondary outcomes that further characterize SWE’s diagnostic utility.

## 2. Materials and Methods

### 2.1. Study Design and Population

**Figure 1:**
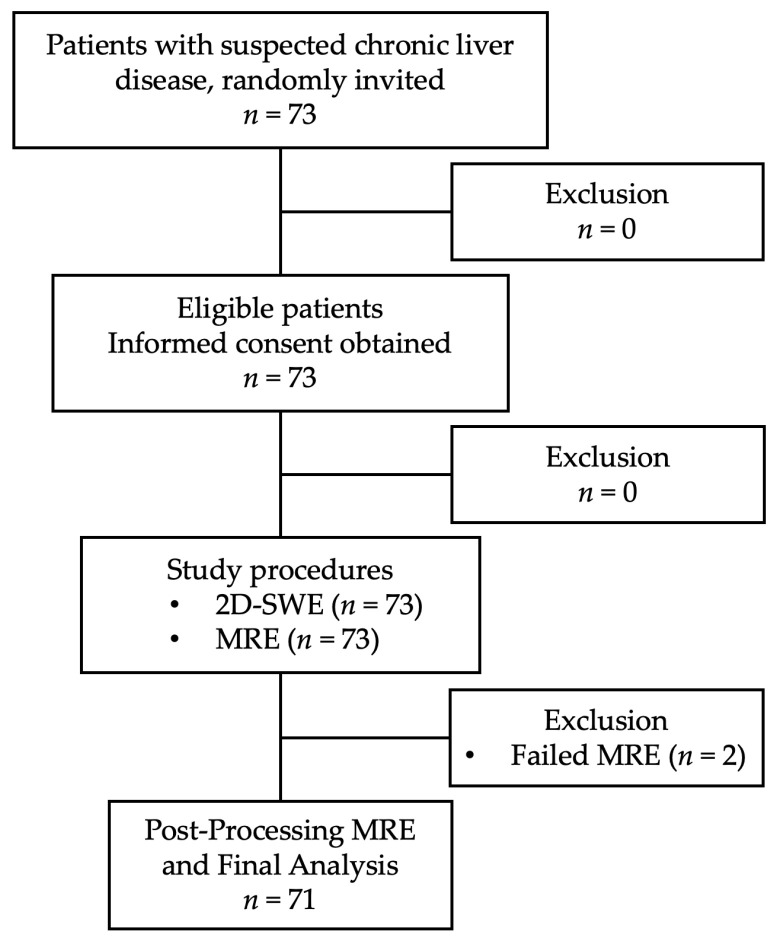
Study flow diagram. Out of 73 eligible patients enrolled, 71 completed both 2D-SWE and MRE imaging. Two patients were excluded due to MRE technical failures, leaving 71 for final analysis.

The study flow diagram is shown in Figure 1; this prospective study included patients referred to the Department of Radiology between November 2022 and September 2024 for ultrasound elastography (US) with suspected chronic liver disease or cirrhosis. Eligible patients were identified according to the inclusion criteria outlined in the study protocol (study code: RAD-2564-08543). The inclusion criteria were as follows: (1) age ≥ 18 years; (2) known or evidence of chronic liver diseases (e.g., abnormal aspartate aminotransferase (AST) or alanine aminotransferase (ALT) levels); (3) ability to lie in a supine position and hold breath; and (4) no contraindication for MRI. Patients who met these criteria were randomly invited to participate and were provided with detailed information about the study procedures, risks, and benefits. All patients gave written informed consent in compliance with the protocol approved by the Faculty of Medicine Ethics Committee (EC) number 353/2022.

### 2.2. Ultrasound Elastography

Ultrasound elastography was conducted using a LOGIQ™ E10 ultrasound machine (GE Healthcare, Chicago, IL, USA), equipped with 2D shear-wave elastography (SWE) capabilities. The imaging was conducted using a GE curvilinear (C1-6) transducer with a frequency range of 1–6 MHz. A fixed frequency of 4.5 MHz was used in all cases to maintain consistency in liver stiffness measurements. The protocol followed the guidelines from the Society of Radiologists in Ultrasound (SRU) and the World Federation for Ultrasound in Medicine and Biology (WFUMB) to ensure standardized and reliable liver stiffness measurements [27,28].

Patients were instructed to fast for 4–6 h prior to the ultrasound examination to minimize postprandial effects on liver stiffness measurements. During imaging, patients were positioned supine or seldomly in a slight left lateral decubitus position with the right arm raised to increase the intercostal space for optimal liver access.

Measurement acquisition: SWE measurements were acquired intercostally with the transducer held perpendicular to the liver capsule. Regions of interest (ROIs) were placed approximately 1.5 to 2.0 cm below the liver capsule, avoiding large blood vessels, shadows, or reverberation artifacts. Measurements were recorded during end-expiration breath holds to reduce motion artifacts.

Quality assurance: Ten measurements were obtained per patient, with measurement validity ensured by maintaining an interquartile range to median (IQR/M) ratio of ≤30%.

To ensure the reliability and reproducibility of 2D-SWE as a method for assessing liver stiffness across varying operator skill levels, a preliminary reproducibility study was conducted within our department. In this mini-study, 34 patients underwent ultrasound elastography performed by both in-training residents and an experienced radiologist (P.C.). The measurements were independently validated by the radiologist, who was blinded to the residents’ results. The results demonstrated a high correlation (r = 0.9375, *p* < 0.001) and a high degree of interobserver agreement (Figure S1). The intraclass correlation coefficient (ICC) was 0.93 (95% CI: 0.87–0.97), indicating strong reliability in liver stiffness measurements between readers. This consistency reinforces the robustness of 2D-SWE as a noninvasive modality suitable for general and abdominal radiologists, as well as those in training. Consequently, cross-measurement by multiple operators was omitted in the main study, with confidence in the reproducibility of 2D-SWE measurements.

### 2.3. Magnetic Resonance Elastography (MRE)

After ultrasound elastography, each patient underwent MRE within two hours for direct cross-modality comparison. MRE was performed using the SIGNA™ Pioneer 3.0T MRI system (GE Healthcare, Chicago, IL, USA) with the integrated MR Touch software (introduced in 2009) for postprocessing of the liver stiffness data. The protocol was designed to align with the Quantitative Imaging Biomarkers Alliance (QIBA) standards for hepatic MRE [29].

Patient preparation and positioning: Consistent with the preparation for ultrasound elastography, patients fasted for 4–6 h before MRE. During MRE, patients were positioned supine, feet first, in the scanner bore. To minimize respiratory motion artifacts, patients were instructed to hold their breath at the end of expiration during image acquisition.

Driver system and frequency: Mechanical vibrations were generated by an external driver set at a frequency of 60 Hz, as per the standard liver MRE protocol [29]. The driver power was adjusted to 50–60% to achieve optimal shear-wave propagation through the liver. The passive driver was secured over the right lower chest wall, aligned with the midclavicular line, to ensure effective energy transfer.

#### The Acquisition Parameters Were as Follows

- Sequence: Echo-planar imaging (EPI) was employed for fast acquisition, reducing susceptibility to motion artifacts.
- Repetition time (TR): 1000 ms.
- Echo time (TE): Minimum full TE, approximately 55.4 ms.
- Flip Angle: 90°.
- Bandwidth: 250 kHz.
- Number of excitations (NEX): 1.
- Field of view (FOV) and matrix: The FOV was set to 420 mm with a matrix of 96 × 96, ensuring adequate coverage and spatial resolution.
- Slice positioning and thickness: Four axial slices, each 8 mm thick with a 2 mm gap, were positioned to capture the liver’s largest cross-sectional area, avoiding the dome and tip for consistent stiffness measurements.

After each patient underwent MRE, the technologists promptly checked the quality of the MRE data to ensure it met our standards. To minimize potential bias, the MRE images were not processed immediately. Instead, they were processed by a single investigator (P.C.) approximately two weeks after data acquisition. This delay helped to prevent the recall of individual SWE results, thus reducing the risk of bias in the analysis. Additionally, the MRE postprocessing was conducted on a GE Advantage workstation (GE Healthcare, Chicago, IL, USA), which was separate from the hospital’s primary Picture Archiving and Communication System (PACS). This separation ensured that the investigator had no access to the SWE results during the MRE image analysis, allowing for an objective interpretation of MRE stiffness values independent of prior SWE findings. Quality assessment measures were implemented to ensure technical success. This included the visual confirmation of the mechanical waves in the phase images and the exclusion of areas with low-confidence elastograms or incoherent wave patterns. The regions of interest (ROIs) encompassed the largest possible area of liver parenchyma, while avoiding areas near the liver margin or large vessels. Liver stiffness was determined by calculating the average stiffness value across multiple valid slices.

In this study, MRE liver stiffness measurements were interpreted using established threshold values to differentiate stages of liver fibrosis. According to recommended guidelines [13,14,15,16,30,31], a liver stiffness value of less than 2.5 kPa indicates normal liver stiffness, while values between 2.5 and 3.0 kPa suggest either a normal liver or potential inflammation. For early fibrosis (Stage 1–2), the value ranges from 3.0 to 3.5 kPa. Significant to advanced fibrosis (Stage 2–3) is represented by values between 3.5 and 4.0 kPa, whereas advanced fibrosis to cirrhosis (Stage 3–4) is indicated by measurements from 4.0 to 5.0 kPa. Finally, a stiffness value exceeding 5.0 kPa is associated with cirrhosis (Stage 4). These cut-offs facilitate a structured approach for assessing liver fibrosis severity using MRE.

### 2.4. Data Collection and Clinical Variables

Demographic and clinical data, including liver function tests, were collected within one month of imaging. Additional clinical data, such as underlying conditions, were recorded to provide context for liver stiffness measures obtained from both 2D-SWE and MRE modalities. These variables were collected for comprehensive analysis and cross-modality comparison.

### 2.5. Statistical Analysis

The sample size estimation was conducted using the Buderer method [32] for diagnostic accuracy studies, based on previous meta-analysis data covering a broad range of liver fibrosis etiologies [33]. With an expected sensitivity of 90% for 2D-SWE and a prevalence of significant fibrosis (≥F2) at 54%, a minimum of 64 patients were required to achieve sufficient statistical power. Accounting for a 20% technical failure rate, we aimed for a total of 80 patients.

Descriptive statistics were calculated for all demographic and clinical variables. Diagnostic accuracy indices including sensitivity, specificity, positive predictive value (PPV), negative predictive value (NPV), positive likelihood ratio (LR+), diagnostic accuracy, and area under the receiver operating characteristic curve (AUROC) were calculated for various liver stiffness cut-off values. ROC analysis was used to evaluate the performance of SWE in diagnosing significant fibrosis (≥F2) and other stages, with MRE as the reference standard. Correlation analysis was conducted to compare stiffness values between SWE and MRE, with the Pearson correlation coefficient and 95% confidence intervals calculated. All statistical analyses were performed using STATA software (version 16.0, StataCorp LLC, College Station, TX, USA), with significance set at *p* < 0.05.

## 3. Results

### 3.1. Patient Characteristics

In this study, 73 patients were eligible, with only 2 exclusions due to unsuccessful MRE image acquisition, resulting in 71 patients included in this study.

As detailed in Table 1, the median age of the cohort was 55 years (interquartile range, IQR 47–63), with a slight male predominance (57.8%). The mean body mass index (BMI) was 24.4 kg/m² (standard deviation, SD 3.9), indicating a diverse range of body habitus within the study population.

The predominant etiology for liver disease was hepatitis B virus (HBV), observed in 60.6% of the patients, followed by hepatitis C virus (HCV) (18.3%) and nonalcoholic fatty liver disease (NAFLD) (15.5%). Notably, 14.1% of patients had combined etiologies, such as concurrent HBV infection with alcohol use or HBV with NAFLD. Other etiologies made up 7% of the cases, reflecting the clinical diversity within the cohort.

Laboratory findings showed a median platelet count of 204 × 10^3^/µL (IQR 83 × 10^3^) and a median serum albumin level of 4.3 g/dL (IQR 0.5). Liver function tests indicated a median aspartate aminotransferase (AST) level of 26 U/L (IQR 13) and an alanine aminotransferase (ALT) level of 25 U/L (IQR 25), suggesting variable levels of hepatic inflammation among the participants.

### 3.2. Relationship Between Liver Stiffness Measurement by 2D-SWE and MRE

**Figure 2:**
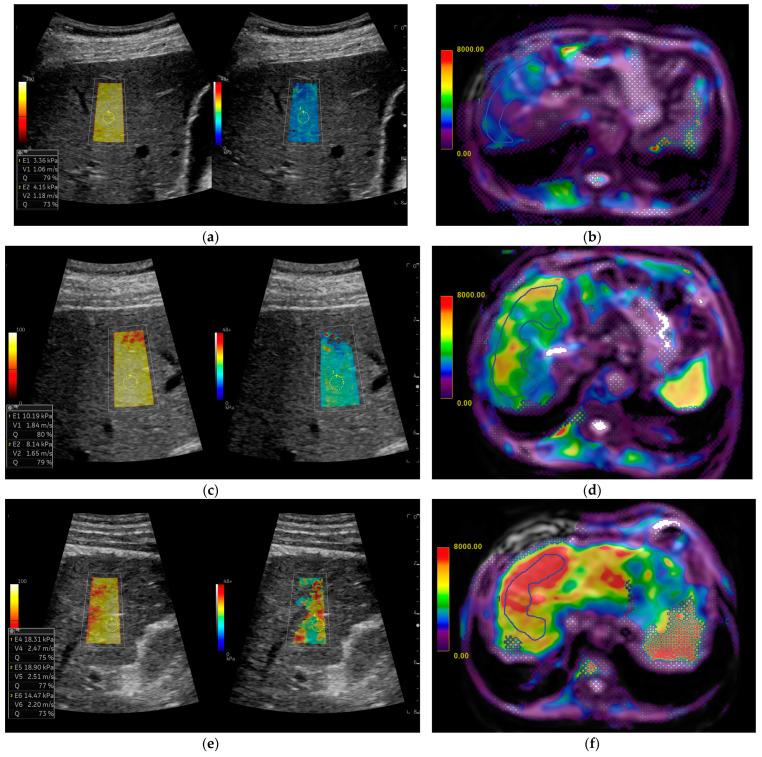
Comparison of liver stiffness using 2D shear-wave elastography (SWE) and magnetic resonance elastography (MRE) across fibrosis stages: (**a**,**b**) normal liver stiffness, with low values in (**a**) 2D-SWE and (**b**) MRE; (**c**,**d**) intermediate fibrosis; and (**e**,**f**) cirrhosis, F4, with high stiffness in both 2D-SWE and MRE. Each pair (**a**-**b**, **c**-**d**, **e**-**f**) represents the same patient at different fibrosis stages. ROIs are also shown in the images as stiffness measurements.

In each patient, liver stiffness measurements were obtained using both 2D-SWE and MRE, as shown in Figure 2.

**Figure 3:**
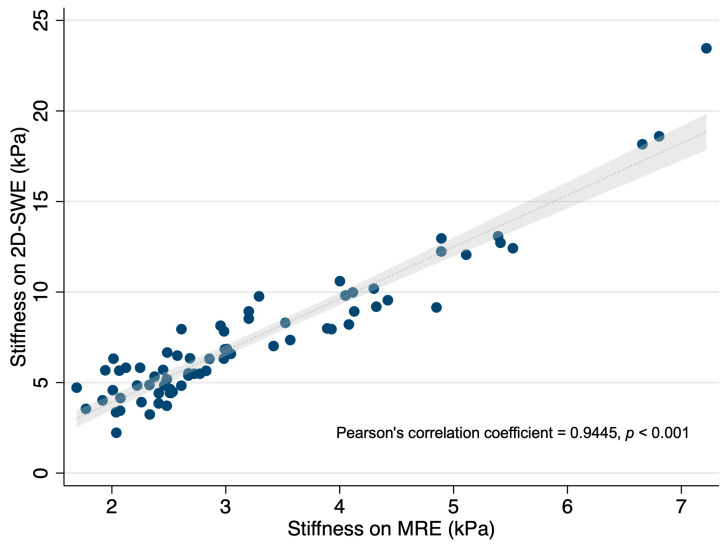
Scatter plot showing the relationship between liver stiffness measurements by 2D-SWE and MRE. Each point represents an individual patient’s stiffness measurement, with stiffness on 2D-SWE (kPa) plotted against stiffness on MRE (kPa). The plot demonstrates a strong positive correlation, with a Pearson correlation coefficient of 0.9445 (*p* < 0.001), suggesting excellent agreement between the two modalities.

A strong positive correlation was observed between the liver stiffness measurements obtained by 2D-SWE and MRE. In Figure 3, the scatter plot demonstrates a close alignment of the stiffness values between the two modalities across the spectrum of stiffness levels. The Pearson correlation coefficient was 0.9445 (*p* < 0.001), indicating a very high degree of agreement.

**Figure 4:**
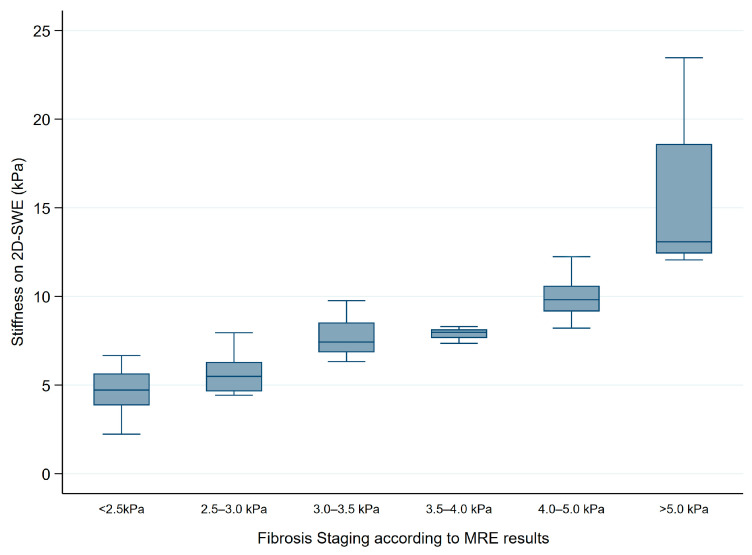
Distribution of 2D-SWE stiffness values across fibrosis stages categorized by MRE cut-off values. MRE thresholds used for fibrosis staging include <2.5 kPa for Normal, 2.5–3.0 kPa for Normal or Inflammation, 3.0–3.5 kPa for Stage 1–2 fibrosis, 3.5–4.0 kPa for Stage 2–3 fibrosis, 4.0–5.0 kPa for Stage 3–4 fibrosis, and >5.0 kPa for Stage 4 fibrosis or cirrhosis. The figure illustrates a progressive increase in median 2D-SWE values corresponding to advancing fibrosis stages.

Figure 4 illustrates the distribution of 2D-SWE stiffness values across the MRE-defined fibrosis stages. The median 2D-SWE values increase with fibrosis severity, from low stiffness in the “Normal” and “Normal or Inflammation” groups to higher values in early (F1–2), intermediate (F2–3), and advanced (F3–4) fibrosis stages, reaching the highest levels in cirrhosis (F4), with notable variability. This trend reinforces the role of 2D-SWE in effectively distinguishing between fibrosis stages.

### 3.3. Diagnostic Accuracies of 2D-SWE for Diagnosing Significant Fibrosis (≥ F2) and Optimal Cut-Off Value

The diagnostic accuracy of 2D-SWE for detecting significant fibrosis (≥F2) was assessed using MRE as the reference standard, defining significant fibrosis as a liver stiffness measurement of ≥3.5 kPa on MRE. Table 2 summarizes the sensitivity, specificity, AUROC, and likelihood ratios for the various 2D-SWE cut-off values.

At a 2D-SWE threshold of 7.0 kPa, sensitivity reached 100%, correctly identifying all cases of significant fibrosis, with a specificity of 85.7%, a positive likelihood ratio (LR+) of 7.0, and an AUROC of 0.93. Increasing the threshold to 8.0 kPa improved specificity to 91.8% while maintaining a sensitivity of 86.4%. This higher threshold achieved an AUROC of 0.89 and an LR+ of 10.58, offering stronger diagnostic specificity.

### 3.4. Assessment of Liver Stiffness for Normal and Other Fibrosis Stages by 2D-SWE

The diagnostic performance of 2D-SWE was assessed for both normal liver stiffness and various stages of liver fibrosis, using MRE as the reference standard. For identifying normal liver stiffness, an MRE cut-off of 2.5 kPa was utilized. At a 2D-SWE threshold of 5.0 kPa, the sensitivity was 89.1%, effectively capturing a high proportion of patients with normal liver stiffness. Increasing the 2D-SWE cut-off to 5.5 kPa resulted in a slight decrease in sensitivity to 82.6% but improved specificity to 72.0%, as outlined in Table 2.

For fibrosis staging, the different 2D-SWE thresholds showed strong diagnostic accuracy across fibrosis stages. A 2D-SWE cut-off of 6.0 kPa yielded 100% sensitivity and 84.6% specificity for detecting mild fibrosis (≥F1, with an MRE threshold of ≥3.0 kPa). For significant fibrosis (≥F2), a threshold of 7.0 kPa provided a robust balance of sensitivity and specificity. For advanced fibrosis (≥F3), a 2D-SWE cut-off of 9.0 kPa achieved high diagnostic accuracy, with increased specificity and likelihood ratios. Lastly, for cirrhosis (≥F4, defined by an MRE threshold of ≥5.0 kPa), a 2D-SWE cut-off of 11.0 kPa provided excellent sensitivity and specificity. The full diagnostic performance metrics for each stage are detailed in Table 2.

## 4. Discussion

This study demonstrates that 2D-SWE is a reliable, noninvasive, and reproducible method for assessing liver stiffness. Its diagnostic accuracy is comparable to that of MRE for staging liver fibrosis. MRE, considered the reference standard for fibrosis evaluation, offers high diagnostic precision without the risks associated with biopsy, particularly in settings where minimizing patient invasiveness is crucial [3,13,14,15,16,34]. Our findings align with those of previous studies that supported the robustness of MRE for comprehensive liver stiffness assessment because it can evaluate stiffness across a broader region of the liver. Despite its advantages, the measurement of liver stiffness using MREs is subject to various confounding factors, including outflow obstruction, inflammation, and biliary obstruction. Additionally, obesity poses limitations to this technique [35]. Liver biopsy remains the gold standard for assessing the extent of fibrosis due to its ability to provide a direct visualization of the actual amount of fibrosis.

The very high correlation observed between 2D-SWE and MRE measurements in this study, as evidenced by a Pearson correlation coefficient of 0.9445, may reflect the similar principles underlying these two elastography techniques. Both 2D-SWE and MRE assess liver stiffness by measuring the propagation of mechanical waves through liver tissue and calculating wave velocity, which correlates with tissue stiffness [36,37,38,39,40,41,42]. This methodological similarity likely contributes to their high concordance in stiffness measurements across varying stages of fibrosis. However, certain confounding factors may influence the liver stiffness values in both techniques, potentially affecting their diagnostic accuracy. For instance, conditions such as hepatic congestion, inflammation, and fatty infiltration can increase liver stiffness independent of fibrosis, which could lead to the overestimation of fibrosis stages in both 2D-SWE and MRE [16]. This highlights a potential limitation of using MRE as a reference standard, as both techniques may be susceptible to similar nonfibrotic influences. Therefore, while MRE remains a strong reference standard due to its reproducibility and whole-liver assessment capability, its susceptibility to nonfibrotic confounders should be considered when interpreting correlation and diagnostic accuracy findings.

In this study, we used a 3.5 kPa cut-off on MRE to define significant fibrosis (≥F2), a threshold supported by established research. This cut-off is well aligned with findings indicating that MRE provides reliable separation between mild and significant fibrosis stages, facilitating noninvasive and accurate fibrosis staging [43]. For example, two studies demonstrated a general progression in liver stiffness values corresponding to fibrosis severity, supporting the use of approximately 3.5 kPa as an effective threshold for distinguishing mild from significant fibrosis stages. [8,44]. Utilizing this 3.5 kPa cut-off as a benchmark in our study strengthens the validity of 2D-SWE’s diagnostic performance, offering a sound reference standard to evaluate fibrosis stages noninvasively.

In detecting significant fibrosis (≥F2), a 2D-SWE threshold of 7.0 kPa achieved 100% sensitivity, affirming its potential for initial screening, as it effectively minimizes false-negative results. Additionally, by increasing the 2D-SWE threshold to 8.0 kPa, specificity rose to 91.8%, enhancing its capacity for high-accuracy fibrosis confirmation. This balance between sensitivity and specificity at different cut-offs suggests that 2D-SWE could be effectively tailored as a screening and diagnostic tool depending on the clinical setting. Similar findings have been reported in other studies, where optimized 2D-SWE cut-offs achieved high sensitivity and specificity for fibrosis stages [23,45].

For ruling out abnormal liver stiffness, a 2D-SWE threshold of 5.0 kPa showed high sensitivity (89.1%) relative to an MRE cut-off of 2.5 kPa. This sensitivity aligns with that in previous studies, confirming the accuracy of 2D-SWE in distinguishing normal from fibrotic liver states, a critical function in population-level screening or early diagnostic settings [27,28]. An increase to 5.5 kPa resulted in slightly reduced sensitivity but retained strong diagnostic accuracy, providing a suitable alternative cut-off that balances sensitivity and specificity.

The results presented here indicate that 2D-SWE’s optimal cut-offs can provide tailored diagnostic solutions: a 7.0 kPa threshold for broad screening and an 8.0 kPa cut-off for significant specificity-driven fibrosis confirmation. These findings suggest that 2D-SWE could complement or, in some cases, be substitute for MRE in clinical settings where MRE access is limited, providing accessible, noninvasive liver stiffness assessment for chronic liver disease management.

For diagnosing cirrhosis (F4), our study demonstrated that a 2D-SWE cut-off of 11.0 kPa yielded the highest diagnostic accuracy, with a sensitivity of 100%, a specificity of 96.9%, and an AUROC of 0.98. This cut-off also achieved an impressive positive likelihood ratio (LR+) of 32.00, confirming its ability to reliably identify cirrhosis, corresponding with the previous studies using various types of elastography [46,47].

MRE is a reliable reference standard for liver fibrosis staging, as supported by previous studies demonstrating its diagnostic precision and consistency across various liver disease etiologies [3,11,48,49]. Its whole-liver assessment capability minimizes the variability caused by sampling constraints and enables accurate fibrosis staging even in patients with conditions like obesity or ascites, where other noninvasive methods may face limitations. In this study, MRE served as a benchmark to validate the performance of SWE.

This study has certain limitations that should be acknowledged. First, while the sample size was sufficient for analysis, a larger, multicenter cohort would enhance the generalizability of our findings across diverse patient demographics and liver disease etiologies. Another limitation is the variability in 2D-SWE values that can arise from different ultrasound system manufacturers, potentially impacting the reproducibility of our results across different equipment. The standardization of 2D-SWE parameters and calibration across devices is essential to ensure consistent diagnostic accuracy. However, recent findings, such as those by Cassinotto et al. [50], suggest that variability between machines is minimal at lower and moderate stiffness ranges, which are clinically relevant for most fibrosis staging, reducing concerns about interplatform discrepancies. 

Ultrasound-based elastography, including 2D-SWE, is subject to confounding factors. Obesity, often cited as a limitation, can affect acoustic wave propagation due to excess subcutaneous fat. However, recent advancements in transducer technology have improved diagnostic accuracy in both patients with and without obesity, mitigating this limitation [51]. Additionally, studies indicate that 2D-SWE is more reliable than TE for assessing liver stiffness in patients with ascites, further demonstrating its utility in challenging clinical scenarios [19]. Inflammation, a common feature of chronic liver disease, can also confound liver stiffness measurements. It increases both the elasticity and viscosity of liver tissue, potentially leading to the overestimation of fibrosis severity [52]. Integrating elastography findings with clinical evaluations is critical for accurate interpretation. Although the current study excluded patients with ascites, future advancements such as viscosity imaging, which may distinguish fibrosis from inflammation, could provide additional diagnostic insights [53]. While 2D-SWE shows promise, significant challenges may still arise in cases of severe obesity or massive ascites, underscoring the need for tailored diagnostic strategies to address these limitations effectively. Finally, while MRE served as an effective reference standard in this study, its accessibility remains limited due to cost and resource requirements, emphasizing the value of accessible alternatives like 2D-SWE [25,26].

The reliability of 2D-SWE measurements was supported by a preliminary reproducibility study conducted within our department, which demonstrated excellent interobserver agreement between an experienced radiologist and residents (ICC = 0.93). This finding reinforces the robustness of 2D-SWE in diverse operator settings, as it showed minimal variability in measurements, even with different levels of operator expertise. However, this study did not specifically address intraobserver reproducibility or machine-based variability, which are important aspects of reliability. The existing literature suggests that reproducibility across ultrasound systems and operators is influenced by variations in hardware, software, and measurement protocols, particularly at higher stiffness ranges [50]. Nevertheless, under standardized protocols, 2D-SWE has demonstrated good reproducibility, especially in the clinically relevant low and moderate stiffness ranges, where most fibrosis staging occurs [46]. Future studies incorporating repeated measurements and multicenter designs could better evaluate intraobserver and cross-device reproducibility, ensuring further validation of 2D-SWE as a reliable tool for liver stiffness assessment in routine practice.

## 5. Conclusions

This study validates 2D-SWE as a reliable, noninvasive, and reproducible method for staging liver fibrosis, with MRE serving as the reference standard. Thresholds of 7.0 kPa for screening significant fibrosis, 8.0 kPa for confirmation, and 11.0 kPa for diagnosing cirrhosis exhibit high diagnostic accuracy. Furthermore, a 5.0 kPa cut-off effectively excludes abnormal liver stiffness, underscoring 2D-SWE’s practicality and accessibility in routine clinical settings.

## Figures and Tables

**Table 1:** Patient characteristics.

Characteristic	*n* = 71		
Median age in years, median (range)	55 (23–74)		
Sex, *n* (%)			
Male	41 (57.8)		
Female	30 (42.3)		
Weight (kg), mean (SD)	63.6 (11.8)		
Height (cm), mean (SD)	161.4 (7.7)		
Body mass index (kg/m^2^), mean (SD)	24.4 (3.9)		
Diabetes or IFG, n (%)	16 (22.9)		
Etiologies, n (%)			
Hepatitis B	43 (60.6)		
Hepatitis C	13 (18.3)		
NAFLD	11 (15.5)		
Alcohol	8 (11.3)		
Iron deposition disease	4 (5.6)		
Autoimmune hepatitis	1 (1.4)		
Platelet count (×10^3^/µL), mean (SD)	204.0 (83.4)		
Albumin (g/dL), mean (SD)	4.3 (0.5)		
Cholesterol (mg/dL), mean (SD)	179.8 (51.1)		
AST (U/L), median (IQR)	26 (22–35)		
ALT (U/L), median (IQR)	25 (17–46)		
Total bilirubin (mg/dL), median (IQR)	0.64 (0.46–0.87)		
Direct bilirubin (mg/dL), median (IQR)	0.27 (0.18–0.40)		
Prothrombin time (PT) (seconds), median (IQR)	11.6 (10.6–12.5)		
Partial thromboplastin time (PTT) (seconds), median (IQR)	32.1 (30.9–34.8)		
International normalized ratio (INR), median (IQR)	1.05 (1–1.17)		
Liver stiffness measurement, median (IQR)	*n* (%)	Modulus (kPa)	Speed (m/s)
Normal	25 (35.2)	4.72 (3.85–5.66)	1.25 (1.13–1.37)
Normal or inflammation	14 (19.7)	5.49 (4.64–6.31)	1.35 (1.24–1.45)
F1-2	10 (14.1)	7.43 (6.84–8.53)	1.58 (1.51–1.69)
F2-3	4 (5.6)	7.97 (7.65–8.15)	1.63 (1.6–1.65)
F3-4	11 (11.5)	9.81 (9.15–10.6)	1.81 (1.75–1.88)
F4	7 (9.9)	13.08 (12.42–18.6)	2.09 (2.03–2.49)

**Table 2:** Diagnostic performance of 2D-SWE in liver fibrosis staging using MRE as the reference standard.

Fibrosis Stage	Cut-Off (kPa)	AUROC	Accuracy	Sensitivity	Specificity	Positive LR	PPV	NPV
Normal vs. abnormal	5.0	0.77 (0.66–0.87)	80.3	89.1 (76.4–96.4)	64.0 (45.5–82.0)	2.48 (1.45–4.22)	82.0 (68.6–91.4)	76.2 (52.8–91.8)
	5.5	0.77 (0.67–0.88)	78.9	82.6 (52.5–92.2)	72.0 (50.6–87.9)	2.95 (1.55–5.61)	84.4 (70.5–93.5)	69.2 (48.2–85.7)
F1-2 MRE ≥ 3.0 kPa	6.0	0.92 0.87–0.98)	91.5	100 (89.1–100)	84.6 (69.5–94.1)	6.50 (3.11–13.57)	84.2 (68.7–94.0)	100 (89.4–100)
	6.5	0.96 (0.91–1.00)	95.8	96.9 (83.8–99.9)	94.9 (82.7–99.4)	18.89 (4.89–72.97)	93.9 (79.8–99.3)	97.4 (86.2–99.9)
F2-3 MRE ≥ 3.5 kPa	7.0	0.93 (0.88–0.98)	90.1	100 (84.6–100)	85.7 (72.8–94.1)	7.00 (3.53–13.90)	75.9 (56.5–89.7)	100 (91.6–100)
	7.5	0.92 (0.85–0.98)	90.1	95.5 (77.2–99.9)	87.8 (75.2–95.4)	7.80 (3.66–16.59)	77.8 (57.7–91.4)	97.7 (88.0–99.9)
	8.0	0.89 (0.81–0.97)	90.1	86.4 (65.1–97.1)	91.8 (80.4–97.7)	10.58 (4.08–27.46)	82.6 (61.2–95.0)	93.8 (82.8–98.7)
	8.5	0.86 (0.76–0.95)	88.7	77.3 (54.6–92.2)	93.9 (83.1–98.7)	12.62 (4.12–38.67)	85.0 (62.1–96.8)	90.2 (78.6–96.7)
F3-4 MRE ≥ 4 kPa	8.5	0.94 (0.88–1.00)	94.4	94.4 (72.7–99.9)	94.3 (84.3–98.8)	16.69 (5.53–50.37)	85.0 (62.1–96.8)	98.0 (89.6–100)
	9.0	0.94 (0.86–1.00)	95.8	88.9 (65.3–98.6)	98.1 (89.9–100)	47.11 (6.71–330.6)	94.1 (71.3–99.9)	96.3 (87.3–99.5)
F4 (cirrhosis) MRE ≥ 5 kPa	10.0	0.97 (0.94–1.00)	94.4	100 (59.0–100)	93.8 (84.8–98.3)	16.00 (6.19–41.32)	63.6 (30.8–89.1)	100 (94.0–100)
	11.0	0.98 (0.96–1.00)	97.2	100 (59.0–100)	96.9 (89.2–99.6)	32.00 (8.18–125.19)	77.8 (40.0–97.2)	100 (94.2–100)
	12.0	0.98 (0.96–1.00)	97.2	100 (59.0–100)	96.9 (89.2–99.6)	32.00 (8.18–125.19)	77.8 (40.0–97.2)	100 (94.2–100)
	13.0	0.79 (0.59–0.98)	95.8	57.1 (11.8–90.1)	100 (94.4–100)	N/A	100 (39.8–100)	95.5 (87.5–99.1)

## Data Availability

The anonymized data generated in this study will be kept confidential in accordance with international research ethics standards and the Personal Data Protection Act B.E. 2562 (2019) of Thailand. The data will be retained for 10 years after the completion of this research project. Although not publicly available, data may be accessible upon official request.

## Supplementary Materials

The following supporting information can be downloaded at: https://www.mdpi.com/article/10.3390/diagnostics15010062/s1, Figure S1: Scatter plot showing interobserver reproducibility of 2D-SWE liver stiffness measurements between residents and an experienced radiologist.
