# Supplementary material for: Two-Dimensional Shear-Wave Elastography: Accuracy in Liver Fibrosis Staging Using Magnetic Resonance Elastography as the Reference Standard
Source: Diagnostics (Basel). 2024 Dec 29;15(1):62. doi: 10.3390/diagnostics15010062 (PMC11719920; doi:10.3390/diagnostics15010062)
Supplement: Supplementary file 1 [file diagnostics-15-00062-s001.zip › diagnostics-3378874-supplementary.pdf]

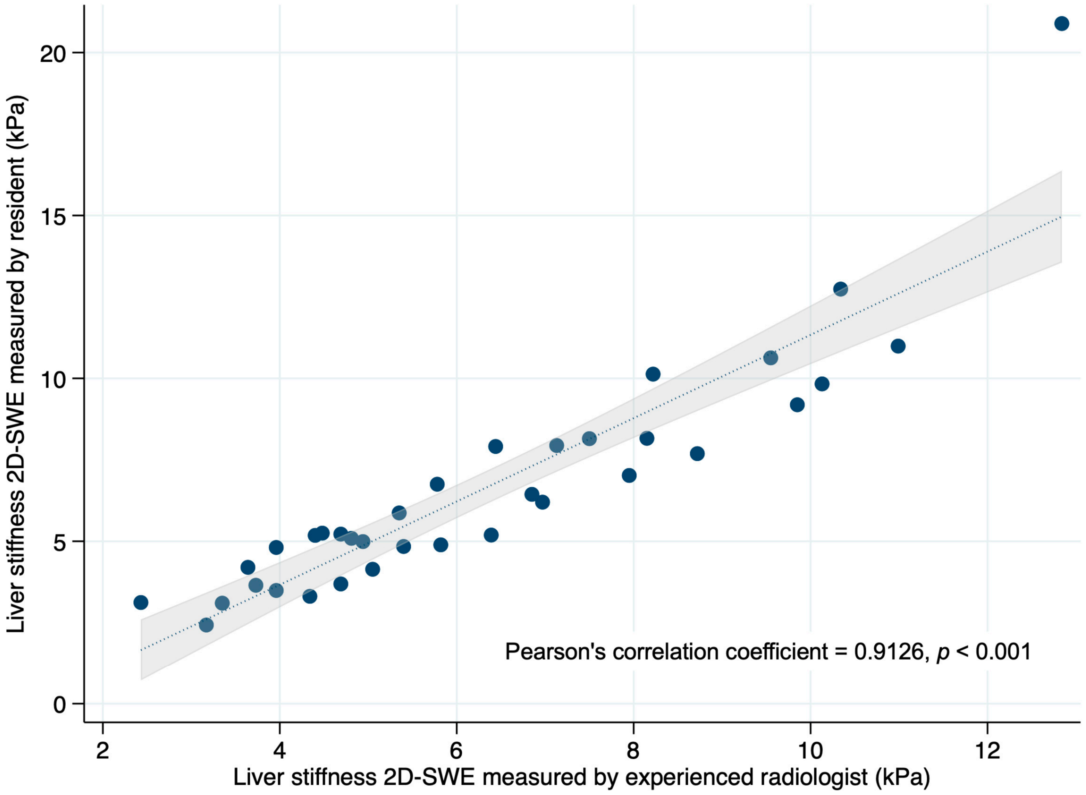

Figure S1: Scatter plot showing interobserver reproducibility of 2D-SWE liver stiffness measurements between residents and an experienced radiologist.
